# Heart Rate Variability, Microvascular Dysfunction, and Inflammation: Exploring the Potential of taVNS in Managing Heart Failure in Type 2 Diabetes Mellitus

**DOI:** 10.3390/biom15040499

**Published:** 2025-03-29

**Authors:** Serge C. Thal, Sergey Shityakov, Ellaine Salvador, Carola Y. Förster

**Affiliations:** 1 Department of Anesthesiology, Helios University Hospital, Witten/Herdecke University, 42283 Wuppertal, Germany; serge.thal@uni-wh.de; 2 Laboratory of Chemoinformatics, Infochemistry Scientific Center, ITMO University, 197101 Saint-Petersburg, Russia; shityakoff@hotmail.com; 3 Section Experimental Neurosurgery, Department of Neurosurgery, University Hospital Würzburg, 97080 Würzburg, Germany; salvador_e@ukw.de; 4 Department of Anesthesiology, Intensive Care, Emergency and Pain Medicine, Section Cerebrovascular Sciences and Neuromodulation, University Hospital Würzburg, 97080 Würzburg, Germany

**Keywords:** type 2 diabetes mellitus, cardiovascular diseases, heart failure, heart rate variability, vagus nerve stimulation, transcutaneous auricular vagus nerve stimulation, autonomic dysfunction, systemic inflammation, microvascular complications

## Abstract

Patients with type 2 diabetes mellitus (T2DM) predominantly experience mortality due to cardiovascular diseases (CVD), particularly in low- and middle-income nations. Among these, heart failure (HF) is the most severe cardiovascular complication in terms of prognosis and management. Despite advancements in individualized glycemic control and cardiovascular risk management, including the development of novel glucose- and lipid-lowering agents, the prevalence of HF in T2DM patients remains persistently high. This indicates that factors beyond hyperglycemia significantly contribute to the heightened risk of HF associated with T2DM. This review examines critical factors influencing CVD risk in T2DM, particularly the roles of reduced heart rate variability (HRV), a marker of autonomic dysfunction, and chronic inflammation, both of which play pivotal roles in HF pathogenesis. Recent evidence highlights the potential of vagus nerve activation to modulate these risk factors, underscoring its capacity to reduce T2DM-related cardiovascular complications. Specifically, we discuss the therapeutic promise of transcutaneous auricular vagus nerve stimulation (taVNS) as a non-invasive intervention to enhance vagal tone, decrease systemic inflammation, and improve cardiovascular outcomes in T2DM. By addressing the interplay among HRV, microvascular disease, and inflammation, this review provides a comprehensive perspective on the potential utility of taVNS in managing HF in T2DM.

## 1. Introduction

Diabetes mellitus (DM) is characterized by chronic hyperglycemia resulting from defects in insulin secretion, insulin action, or both [1]. DM is broadly classified into type 1 (T1DM) and type 2 (T2DM), with additional subtypes including gestational diabetes, maturity-onset diabetes, neonatal diabetes, and secondary forms linked to metabolic syndrome or medication use [2]. T1DM, commonly diagnosed in childhood or adolescence, arises from autoimmune destruction of pancreatic beta cells, leading to absolute insulin deficiency [3]. In contrast, T2DM is defined by progressive insulin resistance and impaired insulin secretion, often resulting from poor lifestyle habits and prolonged hyperglycemia, primarily affecting middle-aged and older adults [1,3]. Due to the distinct pathogenesis of T1DM and T2DM, each subtype exhibits unique etiologies, clinical presentations, and corresponding treatment approaches (reviewed in [4]). Although historically associated with older populations, the prevalence of T2DM has risen rapidly in younger individuals, including children and adolescents, making it a significant global health challenge in the 21st century [5].

Approximately 50% of individuals with T2DM die from cardiovascular diseases (CVD), such as ischemic heart disease, heart failure (HF), stroke, coronary artery disease (CAD), and peripheral artery disease, as well as microvascular complications [6]. Despite advancements in glycemic control and cardiovascular risk management, including newer glucose- and lipid-lowering agents, progress in preventing T2DM-related CVD remains limited. This underscores the need for integrated management strategies to address the growing clinical burden of T2DM-associated CVD.

Recent research has highlighted the role of the vagus nerve in mitigating CVD risk in T2DM [7]. Evidence suggests that vagal activity, which can be assessed through heart rate variability (HRV), is inversely related to diabetes severity, with low HRV serving as a predictor of T2DM [7]. The vagus nerve exerts protective effects by suppressing inflammation through neuroendocrine and neuroimmunological mechanisms. Emerging data indicate that vagal activation may ameliorate diabetes-related pathophysiological processes and biomarkers, although more randomized controlled trials are needed to confirm these effects.

This review focuses on vagal modulation in T2DM, specifically exploring transcutaneous auricular vagus nerve stimulation (taVNS) as a non-invasive strategy to enhance vagal activity, reduce systemic inflammation, and mitigate CVD risk. By addressing the interplay among HRV, microvascular dysfunction, and chronic inflammation, we aim to provide a novel perspective on taVNS as a potential therapeutic intervention in T2DM-associated HF.

## 2. T2DM and Cardiovascular Disease

As T2DM progresses, individuals face a heightened risk of various cardiovascular complications [8]. Beyond conventional CVD risk factors such as hypertension and CAD, T2DM itself is an independent risk factor for cardiomyopathy and heart failure (HF) [9]. The rising prevalence of T2DM worldwide, coupled with an aging population, has significantly contributed to the growing burden of diabetes-related HF [10].

Notably, the increasing recognition of diabetic cardiomyopathy (DCM) highlights that, even in the absence of atherosclerotic CAD, long-term diabetes directly impacts cardiac structure and function. This occurs through mechanisms such as aberrant myocardial metabolism and insulin resistance [11]. DCM and diabetes-related HF exhibit a complex, multifactorial pathophysiology, involving abnormal calcium signaling, impaired glucose and fatty acid metabolism, and inflammatory pathways that drive myocardial fibrosis, stiffness, and hypertrophy [12]. The interplay of these mechanisms often results in asymptomatic diastolic and systolic dysfunction, which may progress to the clinical syndrome of diabetes-related HF.

### 2.1. Diabetic Cardiomyopathy (DCM)

DCM is a distinct subtype of cardiomyopathy characterized by ventricular dysfunction in patients with T2DM, occurring in the absence of CAD, hypertension, or valvular heart disease [13]. It is increasingly recognized as a separate clinical entity, marked by structural and functional abnormalities of the myocardium that are independent of the macrovascular complications commonly associated with T2DM (e.g., hypertension, CAD, and atherosclerosis) [11,14,15]. T2DM-associated DCM can present in varying forms, such as hypertrophic cardiomyopathy [16] or dilated cardiomyopathy [17].

Pathologically, DCM involves alterations in myocardial structure and function, primarily driven by metabolic derangements associated with T2DM. Experimental and clinical studies have linked hyperglycemia, insulin resistance, and lipotoxicity to abnormalities in left ventricular geometry and strain. These metabolic disturbances result in suppressed glucose oxidation, increased free fatty acid metabolism, impaired calcium handling, mitochondrial dysfunction, and oxidative stress [10]. These mechanisms collectively contribute to interstitial and perivascular fibrosis, cardiomyocyte hypertrophy, myocardial stiffness, and reduced ventricular compliance [18,19,20], which underlie both diastolic and systolic dysfunction in DCM [16,17,21].

Beyond structural and functional changes, DCM exhibits a multifactorial and interconnected pathophysiology. In many cases, it coexists with other T2DM-related comorbidities, including atherosclerosis, hypertension, and CAD, complicating its diagnosis and management in patients with HF [22,23]. Furthermore, impaired coronary microvasculature, often observed in DCM patients with T2DM and insulin resistance, exacerbates myocardial dysfunction. Despite advances in understanding DCM, the relative contributions of these mechanisms to the various HF phenotypes in T2DM remain incompletely understood.

### 2.2. T2DM-Related Heart Failure (HF)

HF is a frequent but often underrecognized complication of diabetes [24]. In patients with T2DM, the etiology and severity of DCM and HF vary, with a strong correlation between T2DM and conditions such as CAD, hypertension, and renal disease, all of which increase the risk of DCM and HF [25]. Furthermore, even in the absence of additional HF risk factors, individuals with T2DM experience HF at higher rates [14,26]. Conversely, HF is thought to be a predictor of future T2DM development and is associated with a higher prevalence of T2DM [27].

**Figure 1:**
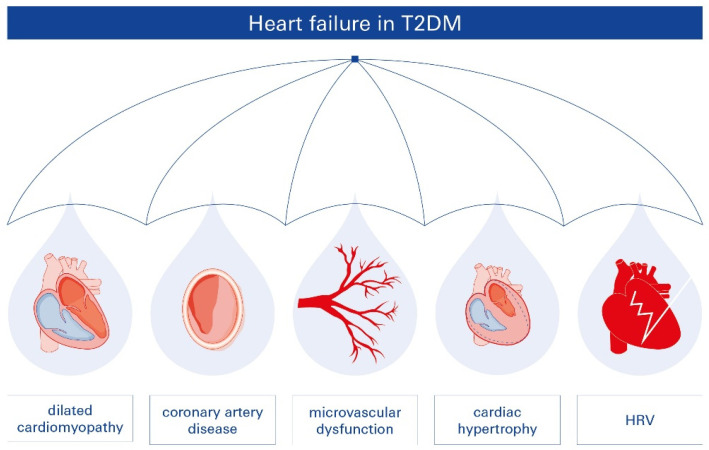
Pathophysiologic connections between T2DM and HF. Diabetes-related HF is multifactorial, presenting with diverse phenotypes and affecting various cardiac structures. Key pathophysiologic mechanisms include microvascular dysfunction, reduced heart rate variability (HRV), coronary artery disease (CAD), dilated cardiomyopathy, and cardiac hypertrophy. These mechanisms interact intricately, contributing to the development and progression of HF in patients with T2DM.

Diabetes-related HF is multifactorial, presenting in various forms and affecting multiple parts of the heart. The pathophysiologic relationship between T2DM and HF involves several interacting mechanisms, including microvascular dysfunction, reduced heart rate variability (HRV), CAD, dilated cardiomyopathy, and cardiac hypertrophy (Figure 1). While diabetic patients often experience ischemic episodes, HF risk increases independently of hypertension or CAD.

The pathophysiology of HF in patients with T2DM is complex, and despite significant progress over the past few decades, many aspects remain poorly understood. Key mechanisms contributing to this condition include enhanced cardiac insulin signaling, glucotoxicity, lipotoxicity, mitochondrial dysfunction, myocardial fibrosis, oxidative stress, impaired cardiac calcium handling, cardiovascular autonomic dysfunction, endocardial dysfunction, and hyperactivation of the renin–angiotensin–aldosterone system (RAAS) [28]. However, the relative contribution of each of these mechanisms to the diabetic cardiomyopathy (DCM) phenotype and their interplay remain unclear.

The type of HF, including heart failure with reduced ejection fraction (HFrEF) or preserved ejection fraction (HFpEF), also influences the underlying pathophysiology of DCM and HF [29]. Importantly, much of the current evidence regarding T2DM-associated HF is derived from animal models. These studies primarily focus on structural abnormalities, metabolic dysfunction, inflammation, and oxidative stress, while also considering gender differences [30]. Although rodent models provide valuable mechanistic insights into diabetes-related heart disease, they fail to fully replicate the complexity of the human condition. Therefore, the development of additional models in other species is essential to advance our understanding of the pathophysiology and clinical progression of diabetes-related heart disease [31]. Recent evidence suggests that HF in T2DM can also be classified as a microvascular disease, a classification that underscores its significant role in increasing morbidity and mortality among diabetic patients.

### 2.3. Microvascular Complications Linked to T2DM

Microvascular dysfunction in patients with T2DM is a major risk factor for adverse cardiovascular events, including ischemic heart disease, HF, stroke, CAD, and peripheral artery disease [32], collectively accounting for at least 50% of deaths among individuals with T2DM [6]. Microvascular complications in diabetic patients are associated with several factors, including age of onset, disease duration, obesity, chronic hyperglycemia, hypertension, and abnormal lipid metabolism [33,34]. Microvascular dysfunction affects multiple organ systems, leading to complications such as diabetic retinopathy (DR), cardiac microvascular disease, and diabetic nephropathy. The presence and severity of microvascular disease significantly influence the progression and prognosis of CVDs in T2DM.

**Figure 2:**
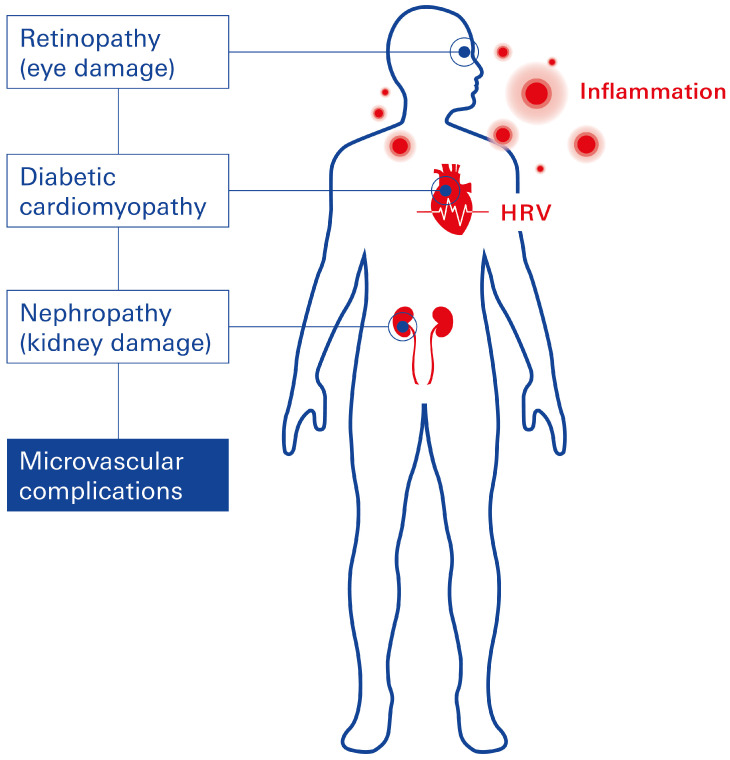
Microvascular complications and endothelial dysfunction in T2D. Microvascular complications in diabetics are similar in etiology, with chronic hyperglycemia initiating metabolic and structural changes associated with the prevalent inflammation and alterations in HRV.

The underlying pathophysiology involves the accumulation of advanced glycation end products (AGEs) within smooth muscle and endothelial cells (ECs) of the myocardium, renal, and retinal microvasculature [35]. AGE deposition triggers vascular inflammation, which in turn reduces endothelial nitric oxide production, leading to impaired vasodilation and vascular dysfunction. Notably, inflammation represents the central pathological hallmark of microvascular complications across all affected organs in T2DM (Figure 2).

#### 2.3.1. Diabetic Retinopathy (DR)

DR has been the most extensively studied complication of T2DM-related microvascular dysfunction. The primary clinical feature of DR is dysfunction of the inner blood-retinal barrier (iBRB), which plays a critical role in maintaining neural homeostasis and protecting neural tissue from bloodborne toxins [26]. This dysfunction is characterized by increased permeability of retinal endothelial cells (ECs) to plasma proteins and fluid. The disruption of the iBRB can be triggered by inflammation, hyperglycemia, and the loss of supporting cells such as pericytes. Additionally, increased levels of growth factors (e.g., vascular endothelial growth factor [VEGF]), cytokines (e.g., tumor necrosis factor-alpha [TNF-α], interleukin-1 beta [IL-1β]), and advanced glycation end products (AGEs) contribute to the pathogenesis. Cytokines, in particular, induce the inactivation and inhibition of contact proteins like claudin-5, leading to the opening of tight junctions, as demonstrated in experimental DR models [36]. This results in increased paracellular and transcellular vesicular transport across the retinal vessel wall. As a consequence, DR is a major cause of blindness in working-age adults [37]. Currently, approximately 25% of diabetic patients worldwide have DR, and the prevalence of vision loss due to DR is projected to rise [38]. A significant link between DR and CVD, one of the leading causes of mortality, has been established. Between 2006 and 2016, global CVD-related deaths increased by 14.5% [39]. Emerging studies show a strong correlation between DR and the onset of CVD in individuals with T2DM [40,41]. Notably, DR and CVD share common risk factors, including hyperglycemia and hypertension, as well as similar pathophysiological mechanisms such as oxidative stress, inflammation, and epigenetic modifications [42,43,44].

#### 2.3.2. Microvascular Dysfunction in the Diabetic Heart

**Figure 3:**
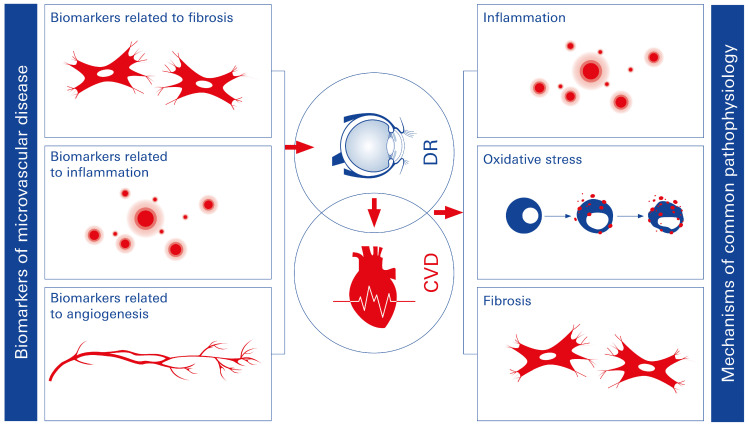
Shared pathophysiological mechanisms between DR and CVD in T2DM. DR and CVD share common risk factors and mechanisms, such as inflammation and oxidative stress. These shared pathways highlight DR’s potential as an early marker for predicting cardiovascular complications in T2DM.

Patients with T2DM and CVD frequently experience endothelial dysfunction, which disrupts endothelial–cardiomyocyte communication and impairs vascular function [45]. Constricted left ventricular remodeling and diastolic dysfunction are closely associated with reduced myocardial nitric oxide bioavailability [46]. Additionally, pericyte loss and capillary rarefaction have been linked to T2DM. Microcirculatory dysfunction can further result in tissue hypoxia, reduced coronary flow reserve, increased myocardial stiffness, and impaired contractility, all contributing to reduced myocardial perfusion [46]. Recent evidence suggests that the pathophysiology of DR and systemic vascular complications in T2DM may share common mechanisms. DR, therefore, may serve as an indicator of systemic microcirculatory disease, affecting not only the eye but also other vital organs, such as the heart and kidneys [47]. Moreover, shared immunometabolic pathways involved in DR and diabetes-related HF facilitate early diagnosis and prediction of DCM. The accessible diagnostic tools in ophthalmology could provide valuable insights into systemic microvascular disorders, highlighting a common pathological basis for end-organ damage in T2DM. Targeting these shared immunometabolic pathways could enhance the effectiveness of therapeutic and preventive strategies for microvascular complications in diabetes. Developing therapies based on these mechanisms and implementing detailed progression analyses could significantly improve both the lifespan and quality of life for affected patients [48,49]. Blood-based biomarker analyses may further aid in the identification and monitoring of disease progression (Figure 3).

#### 2.3.3. Diabetic Nephropathy

Diabetic nephropathy is a significant microvascular complication of T2DM and a major contributor to the excess mortality observed in diabetic patients [50,51]. Nearly one-third of individuals with diabetes develop nephropathy, characterized by proteinuria and microalbuminuria, both of which are strong predictors of cardiovascular risk [52,53,54]. When compared to patients with normoalbuminuria, the relative risk of DCM in individuals with type 1 diabetes is 1.2-fold higher in those with microalbuminuria [55] and 10-fold higher in patients with proteinuria [56]. Microalbuminuria in turn increases the risk of DCM by two- to three-fold [57], while proteinuria increases the risk by nine-fold [58]. An excess of cardiovascular risk factors, such as dyslipidemia, hyperglycemia, and multiple prothrombotic and atherogenic alterations, are linked to the onset of microalbuminuria [59,60]. The underlying pathophysiology involves endothelial dysfunction, renin–angiotensin–aldosterone system (RAAS) activation, and structural defects in vascular basement membranes, all of which exacerbate both renal and cardiovascular complications [61].

### 2.4. Autonomic Dysfunction and Autonomic Neuropathy in T2DM

Patients with T2DM are at increased risk for autonomic nervous system dysfunction, particularly cardiovascular autonomic neuropathy (CAN). CAN affects the sympathetic and parasympathetic components of cardiac innervation, initially presenting as sympathetic overactivation followed by parasympathetic dysfunction [12,62]. This imbalance contributes to the pathogenesis of CVD, including myocardial ischemia, left ventricular (LV) remodeling, and both systolic and diastolic dysfunction [63,64]. In its early stages, CAN is often asymptomatic [65]. However, in advanced stages, it manifests as resting tachycardia, orthostatic hypotension, impaired blood pressure regulation, a blunted heart rate response to exercise, and reduced heart rate variability (HRV). CAN is also associated with an increased risk of cardiac arrhythmias and sudden cardiac death [66]. The primary pathophysiological mechanism of CAN involves chronic hyperglycemia-induced oxidative stress, which leads to neuronal damage, apoptosis, and dysfunction of the sympathetic and parasympathetic nervous systems. HRV, a measure of autonomic nerve function, is significantly reduced in patients with CAN and serves as a key diagnostic tool for early detection of autonomic neuropathy in T2DM [63,66].

### 2.5. Heart Rate Variability (HRV)

HRV reflects the balance between the sympathetic and parasympathetic nervous systems and provides insight into autonomic nervous system function. Reduced HRV is strongly associated with increased cardiovascular mortality, including silent myocardial infarction and arrhythmias, in patients with T2DM [62,67,68,69,70].

**Figure 4:**
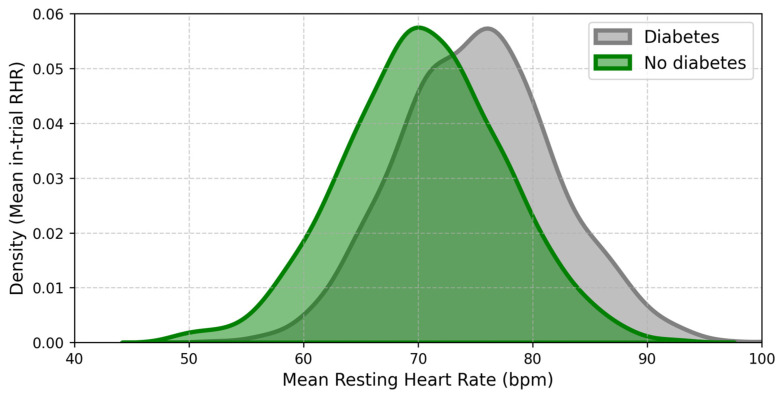
Population density of mean in-trial resting heart rate according to the T2DM and non-T2DM group. The figure compares the population density distribution of mean resting heart rate (RHR) between individuals with T2DM (grey) and those without T2DM (green). The diabetic group shows a higher RHR, suggesting autonomic dysfunction, particularly reduced heart rate variability (HRV). This dysregulation is a known risk factor for cardiovascular complications, including heart failure (HF) in T2DM. These findings emphasize the need for interventions such as transcutaneous auricular vagus nerve stimulation (taVNS) to enhance autonomic function, improve HRV, reduce systemic inflammation, and manage HF in patients with T2DM [72].

HRV measurements demonstrate an inverse relationship between vagal activity and T2DM progression, with low HRV serving as an early predictor of diabetes onset and its complications (Figure 4) [7]. In individuals with T2DM, reduced HRV has also been linked to an elevated risk of sudden cardiac death [71]. Recent evidence suggests that vagal nerve activation may modulate autonomic function and improve HRV, potentially mitigating diabetes-related processes and biomarkers. Abnormal HRV patterns, particularly nonlinear parameters, are frequently observed in patients with metabolic syndrome and T2DM [62]. Additionally, short-term and 24-h electrocardiographic recordings consistently reveal lower HRV variables in patients with diabetes compared to non-diabetic individuals [62]. From a clinical perspective, reduced HRV highlights the need for interventions that enhance vagal activity to restore autonomic balance and reduce cardiovascular risk in T2DM.

In summary, while the exact pathophysiology of diabetes-associated CAN remains unclear, oxidative stress and inflammation induced by chronic hyperglycemia are thought to play a key role in causing neuronal damage and degeneration [62].

### 2.6. T2DM and Chronic Inflammation

Systemic inflammation is a major factor linking CAD, HF, T2DM, and obesity [25]. In obesity, chronic low-grade inflammation is driven by immune cell infiltration into expanding adipose tissue, which results in excessive production of pro-inflammatory cytokines and chemokines [73]. This inflammatory state exacerbates insulin resistance, increases the risk of T2DM, and accelerates the development of diabetes-related complications. Similarly, systemic inflammation is highly prevalent in HF patients, regardless of left ventricular ejection fraction (LVEF), and contributes to disease onset, progression, and poor prognosis [74]. Evidence from animal models highlights a complex interplay between multiple inflammatory pathways in the development of DCM and cardiac inflammation [75]. Furthermore, the inflammatory aspect of diabetes is reported to significantly correlate with the formation of the NOD-like receptor family pyrin domain-containing (NLRP) 3 inflammasome. NLP3 is involved in causing and exacerbating CVD [76,77,78]. This also applies to HF and corneal disease [79,80,81,82]. The most common arrhythmia affecting people worldwide, which is atrial fibrillation (AF), could also be affected by NLRP3, considering that inflammation drives the pathophysiology of AF, as well as obesity, diabetes, hypertension, and HF. This inflammasome has been regarded as one that influences chronic inflammation in the atrial myocardium [83]. Although the pathogenesis of AF is not yet fully elucidated, studies have shown that NLRP3 activation contributes to AF [84,85]. Nonetheless, since there are not enough studies that completely validate this aspect, further investigations are necessary.

Inflammation is a nonspecific tissue response to injury, which involves the recruitment of immune cells and the release of inflammatory mediators. In T2DM, monocytes are recruited to endothelial cells (ECs) via vascular cell adhesion molecule-1 (VCAM-1), facilitated by chemokines such as monocyte chemoattractant protein-1 (MCP-1) and its receptor CCR2. Upon infiltration, monocytes differentiate into macrophages and, in the presence of oxidized low-density lipoprotein (oxLDL), form foam cells, which are key players in atherosclerosis progression [86]. Foam cells secrete inflammatory cytokines, amplifying the local inflammatory response within the lesion site [87]. Matrix metalloproteinases (MMPs), secreted by activated macrophages, degrade the extracellular matrix and collagen fibers within atherosclerotic plaques, promoting their rupture, hemorrhage, and thrombosis [88]. Hyperglycemia-induced metabolic abnormalities further exacerbate inflammation by increasing oxidative stress, mitochondrial ROS production, and activation of the NF-κB pathway. This cascade upregulates inflammatory cytokines, increases vascular permeability, and worsens microvascular complications such as DR and diabetic kidney disease [89].

In proliferative DR, neovascularization is the hallmark pathology. Pro-inflammatory cytokines directly induce angiogenesis by binding to endothelial cells, or indirectly stimulate the production of pro-angiogenic mediators such as vascular endothelial growth factor (VEGF) and angiopoietin-1 (Ang-1). Conversely, these pro-angiogenic factors can upregulate inflammatory cytokine expression in ECs, creating a reciprocal relationship between inflammation and angiogenesis [90]. Inflammation also serves as a key driver of atherosclerosis, the pathological foundation of T2DM-related cardiovascular disease. In its early stages, inflammatory changes in endothelial cells—mediated by blood flow disturbances—trigger leukocyte recruitment and cytokine release [44,91]. Endothelial activation leads to increased expression of adhesion molecules such as intercellular adhesion molecule-1 (ICAM-1) and VCAM-1, along with chemokines like MCP-1. These molecules attract monocytes and lymphocytes, which bind to the endothelium, infiltrate the arterial wall, and initiate plaque development. Among these factors, VCAM-1 plays a particularly central role in the progression of atherosclerosis.

### 2.7. Myocardial Fibrosis

Myocardial fibrosis is a hallmark of T2DM-induced cardiac remodeling and is closely linked to myocardial dysfunction. It is characterized by excessive extracellular matrix (ECM) deposition, including stiff collagen crosslinking, which leads to impaired cardiac compliance and function. A direct correlation has been observed between myocardial fibrosis severity and elevated HbA1c levels in diabetic patients [92]. T2DM-related myocardial fibrosis involves several structural changes, including basement membrane thickening, perivascular fibrosis, microaneurysm formation, and coronary microvascular sclerosis. These alterations are driven by hyperglycemia, hyperinsulinemia, oxidative stress, autonomic dysfunction, inflammation, and overactivation of the renin–angiotensin–aldosterone system (RAAS). At the cellular level, hyperglycemia promotes fibroblast activation and differentiation into myofibroblasts, which excessively produce ECM proteins, primarily collagen types I and III. Advanced glycation end products (AGEs), resulting from chronic hyperglycemia, further stiffen the ECM through non-enzymatic crosslinking of collagen fibers. This process exacerbates diastolic dysfunction by reducing myocardial elasticity and increasing stiffness [12]. Inflammatory cytokines, including TGF-β (transforming growth factor-beta), play a central role in fibrotic remodeling. They stimulate fibroblasts to produce ECM while suppressing ECM degradation. Additionally, oxidative stress and mitochondrial dysfunction contribute to cardiomyocyte apoptosis, which triggers fibroblast activation and perpetuates fibrosis.

## 3. Impact of the Vagus Nerve on T2DM: Exploring the Connection

Emerging evidence suggests that the vagus nerve plays a critical role in both the onset and management of T2DM. Understanding the interplay between vagal function and diabetes could offer novel therapeutic opportunities for this chronic disease [7].

### 3.1. The Vagus Nerve

The vagus nerve is the longest cranial nerve in the human body, extending from the brainstem to the abdomen. It serves as the primary pathway of the parasympathetic nervous system and plays a pivotal role in regulating various physiological processes, including heart rate, gastrointestinal motility, pancreatic function, and immune responses [93,94,95,96]. Through bidirectional communication with peripheral organs, the vagus nerve maintains homeostasis and modulates the autonomic nervous system balance, particularly favoring parasympathetic activity [97]. Beyond its autonomic role, the vagus nerve has anti-inflammatory properties, which are mediated through the cholinergic anti-inflammatory pathway. This pathway involves vagal efferent fibers releasing acetylcholine, which interacts with macrophage nicotinic acetylcholine receptors to inhibit the production of pro-inflammatory cytokines such as TNF-α, IL-1β, and IL-6 [98,99,100].

### 3.2. Diabetic Syndrome and the Vagus Nerve

Dysregulation of vagal activity has been implicated in the pathogenesis of T2DM. Abnormalities in vagal tone, balance, or sensitivity can impair insulin secretion, disrupt glucose homeostasis, and exacerbate metabolic dysfunctions associated with diabetes. For example, diminished vagal activity can contribute to insulin resistance and poor glycemic control—hallmarks of T2DM [101]. Conversely, systemic inflammation, a key characteristic of T2DM, can further impair vagal function, suggesting a reciprocal relationship between vagal tone and metabolic dysregulation [7]. Low vagal activity, as indicated by reduced heart rate variability (HRV), has been identified as a predictive marker for the onset and progression of T2DM [102].

### 3.3. Diabetes Management and Transcutaneous Vagus Nerve Stimulation (tVNS)

The vagus nerve, beyond its possible role in the onset of diabetes, shows significant potential in the management of T2DM. Transcutaneous vagus nerve stimulation (tVNS) is a non-invasive intervention that delivers electrical impulses to stimulate vagal fibers, thereby modulating autonomic and metabolic functions. The auricular branch of the vagus nerve (ABVN), which innervates areas of the external ear such as the cymba conchae and tragus, serves as the primary target for tVNS [103]. The great auricular nerve (GAN), a superficial branch of the cervical plexus, primarily innervates the earlobe, making it a common site for sham control in clinical studies [104]. tVNS has been applied successfully in several conditions, including depression [105,106,107,108,109] and epilepsy [110,111,112]. Its emerging role in diabetes management is particularly promising. Preliminary research indicates that tVNS can reduce systemic inflammation. tVNS stimulates the cholinergic anti-inflammatory pathway, leading to decreased production of pro-inflammatory cytokines (e.g., TNF-α, IL-1β, IL-6), which are major contributors to insulin resistance and chronic inflammation in T2DM [7,113]. tVNS can also improve insulin sensitivity and glucose regulation. By enhancing parasympathetic tone, tVNS may reduce insulin resistance and improve glycemic control. Electrical impulses transmitted via vagal pathways can positively influence pancreatic function and glucose metabolism.

Despite these promising findings, further research is necessary to fully elucidate the clinical benefits and underlying mechanisms of tVNS in diabetes management. Optimization of stimulation parameters, such as frequency, intensity, and duration of electrical impulses, remains a key focus to maximize therapeutic outcomes. Additionally, long-term studies are required to evaluate safety, efficacy, and applicability across diverse patient populations.

#### 3.3.1. The Promising Triad in the Management of T2DM: HRV, Microvascular Disease, and Inflammation

Low heart rate variability (HRV), microvascular dysfunction, and chronic inflammation form an interconnected triad that significantly contributes to the pathogenesis and progression of T2DM and its cardiovascular complications. Recent studies have suggested that transcutaneous auricular vagus nerve stimulation (taVNS) may offer therapeutic benefits by targeting this triad [7].

HRV and taVNS in T2DM. taVNS has emerged as a promising intervention to modulate autonomic function, particularly HRV, which is often reduced in individuals with T2DM.

HRV serves as an indicator of autonomic regulation, influenced by both sympathetic and parasympathetic activity, as well as other physiological factors. Reduced HRV in T2DM is indicative of impaired autonomic regulation and is associated with an elevated risk of cardiovascular complications, including heart failure and sudden cardiac death.

Mechanism of Action. taVNS modulates both peripheral and central neurophysiology. By stimulating afferent fibers of the auricular branch of the vagus nerve (ABVN), taVNS activates brainstem centers, which, in turn, enhance parasympathetic efferent activity. This process increases the release of acetylcholine onto the heart’s sinoatrial node, resulting in a reduction of heart rate and an increase in vagal-mediated HRV. HRV parameters, such as high-frequency power (HF, 0.15–0.40 Hz) and short-term beat-to-beat variations (e.g., root mean square of successive differences), serve as indirect markers of vagal tone and parasympathetic activity [114].

HRV Modulation by taVNS. Several studies have demonstrated the efficacy of taVNS in improving HRV. A recent investigation comparing taVNS stimulation at the cymba conchae with sham stimulation at the helix reported significant increases in HRV frequency-domain parameters, indicating vagal cardiac effects. Specifically, a lower low-frequency to high-frequency (LF/HF) ratio was observed, reflecting a shift toward parasympathetic dominance [114]. Notably, as the auricular branch lacks direct cardiac efferents, these effects are likely mediated through central autonomic pathways. Stimulation patterns play a critical role in determining efficacy. For example, a study comparing biphasic and triphasic burst stimulation patterns found that while both enhanced cardiac autonomic modulation in healthy individuals, triphasic stimulation achieved comparable effects at lower intensities [115].

The efficacy of taVNS may also vary across patient populations. A recent study investigating the impact of age reported that active taVNS significantly increased the high-frequency component of HRV compared to sham stimulation. Importantly, age emerged as a significant modifier: older individuals exhibited a greater increase in high-frequency HRV following active taVNS, while younger subjects maintained stable high-frequency HRV regardless of stimulation type. These findings suggest that older individuals may benefit more from active taVNS in maintaining or improving parasympathetic tone, whereas younger individuals demonstrate greater autonomic adaptability [116].

Further studies have confirmed that taVNS applied to the cymba conchae elicits more pronounced and rapid increases in HRV parameters compared to stimulation at other auricular sites (101) [113]. While these initial findings are promising, additional research is required to optimize taVNS parameters, including stimulation frequency, intensity, and duration, to maximize therapeutic efficacy. Further investigations involving larger and more heterogeneous cohorts, as well as the incorporation of relevant biomarkers, are critical to establishing taVNS as a reliable, non-invasive intervention for autonomic dysfunction in T2DM patients.

Clinical Implications for T2DM. Further research utilizing heterogeneous patient samples and incorporating relevant biomarkers is essential to confirm and expand upon the observed effects of taVNS. Shorter stimulation durations could be particularly valuable for evaluating the immediate impacts of taVNS on heart rate variability (HRV), a physiological marker of autonomic function with well-established clinical relevance. Additionally, future studies should focus on taVNS-induced changes in brain networks that regulate cardiac activity, which will be instrumental in developing robust clinical trial protocols and deepening the understanding of taVNS’s physiological mechanisms.

HRV is increasingly being recognized as a potential risk marker for the onset of diabetes and a promising management strategy for T2DM [117]. This is particularly significant given that CVD is a prevalent complication of T2DM [118]. Monitoring HRV to track the progression of T2DM could provide valuable insights, as diabetic patients typically exhibit lower HRV compared to non-diabetic individuals. Lower HRV is associated with an increased risk of CVD and higher mortality following acute myocardial infarction [119].

Heart rate variability (HRV)-based therapies are emerging as promising interventions for improving glycemic regulation in diabetes [120]. However, the clinical impact of vagal nerve activation on type 2 diabetes mellitus (T2DM) outcomes remains underexplored. While substantial evidence supports the association between HRV and T2DM, direct investigations into the effects of vagal stimulation are limited. Recently, a multicenter trial evaluated the efficacy of cervical transcutaneous vagus nerve stimulation (tVNS) in managing diabetic gastroenteropathy. This study, which utilized a commercially available stimulation system, found no significant improvements in gastrointestinal symptoms or cardiovascular autonomic parameters in individuals with diabetes and autonomic neuropathy compared to sham stimulation [121]. Importantly, the stimulation site differed from auricular tVNS, which may explain variations in efficacy. Evidence suggests that different stimulation sites and modes can yield divergent effects on HRV modulation [115,122]. Consequently, further research is essential to clarify the specific impacts of auricular tVNS on HRV and its potential therapeutic benefits in T2DM.

Existing investigations include both animal studies and clinical trials exploring HRV biofeedback in T2DM patients [123]. Preclinical models have demonstrated that both electrical vagal nerve stimulation and pharmacological vagomimetic activation improve glucose regulation, likely through enhanced vagal nerve activity [124].

taVNS and Modulation of Inflammation. An emerging application of VNS is its use as an anti-inflammatory treatment, particularly relevant for chronic conditions such as T2DM, where inflammation significantly contributes to disease progression [98,99]. This therapeutic approach is supported by evidence that humoral and neuronal reflex pathways regulate inflammatory processes. Chronic inflammation in T2DM is primarily driven by an imbalance between immune and metabolic processes. Moreover, an imbalance between pro- and anti-inflammatory cytokines is considered a central mechanism in the progression of T2DM [100]. These findings underscore the importance of vagus nerve (VN) activity in defending against infections and inflammation. While the parasympathetic nervous system (PNS) exerts exclusively anti-inflammatory effects, the sympathetic nervous system (SNS) demonstrates both pro- and anti-inflammatory roles. This dichotomy is further highlighted by the inverse relationship between inflammatory markers and parasympathetic HRV indices [125,126].

**Figure 5:**
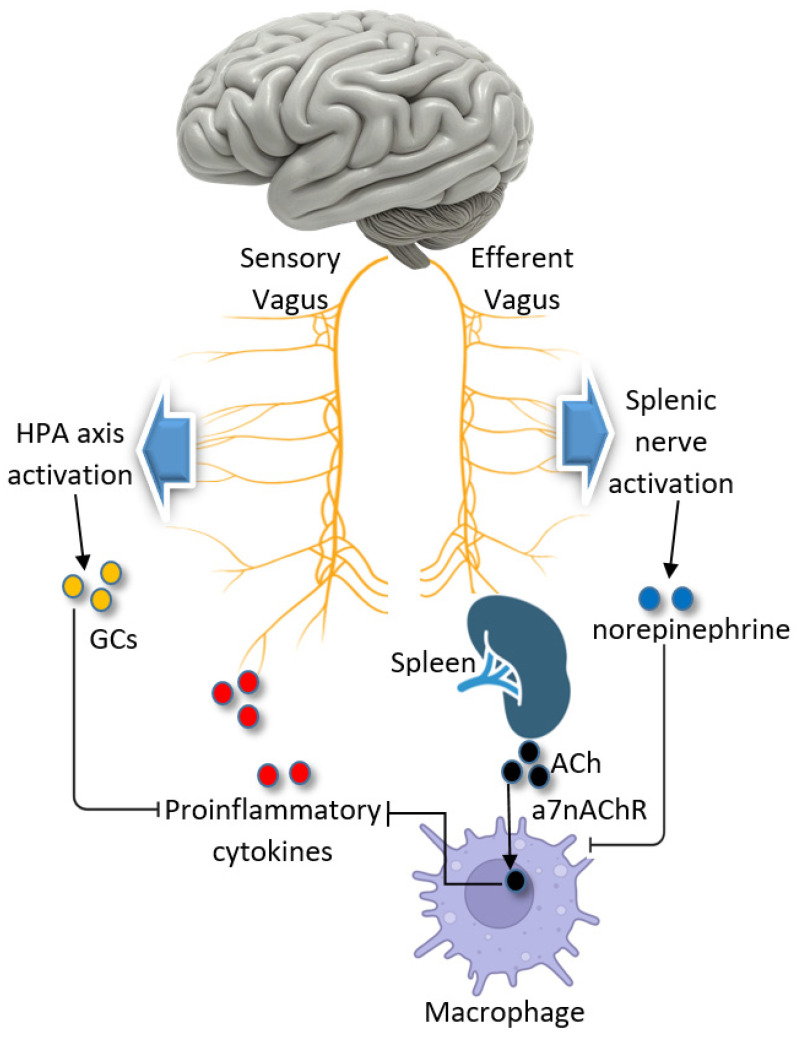
This figure provides a visual representation of the complex interplay between the vagus nerve and systemic inflammation, highlighting the potential therapeutic targets for conditions like T2DM and HF.

Three major reflex pathways involving the VN have been identified as critical to reducing inflammation (Figure 5): the cholinergic anti-inflammatory pathway, the anti-inflammatory vago-vagal reflex, and the anti-inflammatory splenic sympathetic pathway [127,128]. In the cholinergic anti-inflammatory pathway, acetylcholine is released at the synapses of efferent VN fibers when an infection activates the VN. Acetylcholine binds to receptors on macrophages, effectively inhibiting the release of pro-inflammatory cytokines [129]. The anti-inflammatory vago-vagal reflex operates through the hypothalamic-pituitary–adrenal (HPA) axis. Here, infections and injuries activate the nucleus tractus solitarius (NTS) via pro-inflammatory cytokines. This activation triggers a cascade involving the hypothalamus and pituitary gland, ultimately resulting in glucocorticoid release from the adrenal glands. These glucocorticoids play a central role in dampening peripheral inflammation. Finally, in the splenic sympathetic pathway, efferent VN fibers facilitate norepinephrine release from sympathetic nerve terminals in the spleen, which promotes acetylcholine release from lymphocytes. Acetylcholine subsequently suppresses macrophage activity, reducing inflammation [129].

These mechanisms suggest that VN activity is integral to maintaining homeostasis and controlling inflammatory responses. This insight raises critical questions about the potential of VNS to maintain immune balance, prevent immunosuppression, and serve as a treatment for chronic inflammatory diseases. Evidence from recent studies supports the role of VNS in reducing pro-inflammatory markers and preserving homeostasis. Such findings justify expanding the therapeutic use of VNS for T2DM. In a recent study involving T2DM patients, tVNS was demonstrated to improve gastrointestinal motility and reduce inflammatory responses, further underscoring its potential as a novel therapeutic option [130]. Despite these promising findings, additional research is needed to clarify the precise effects of tVNS on circulating cytokines and its clinical relevance in T2DM. Enhanced understanding of these mechanisms will be crucial for optimizing tVNS as a therapeutic intervention and broadening its clinical applications in T2DM and other inflammatory conditions.

taVNS and Microvascular Disease in T2DM. The development of both macrovascular and microvascular complications in T2DM is strongly linked to hyperglycemia. Elevated blood glucose levels lead to chronic metabolic and vascular disturbances that exacerbate the risk of conditions such as DR, nephropathy, and coronary microvascular dysfunction. Additionally, excessive lipid accumulation contributes to insulin resistance, cardiac fibrosis, and diastolic dysfunction in the heart [131,132]. Despite the fragmented and limited body of research, the beneficial effects of transcutaneous vagus nerve stimulation (taVNS) as an adjunct therapy in addressing these complications have been increasingly documented.

Coronary Microvascular Dysfunction (CAD) and taVNS. Emerging evidence suggests that taVNS exerts positive effects on coronary microvascular dysfunction in patients with T2DM. Studies have demonstrated improved coronary microvascular function following taVNS application, primarily through its anti-inflammatory mechanisms [133]. Inflammatory pathways are pivotal in the progression of T2DM-related cardiac dysfunction, and taVNS appears to mitigate these pathways, leading to improved cardiac outcomes [134]. Moreover, recent findings have indicated that taVNS may have broader therapeutic implications, extending its benefits to cardiac fibrosis, thereby making it a potential add-on therapy for managing T2DM-related cardiac complications [135]. However, the need for systematic and large-scale investigations into these effects remains pressing.

DR and taVNS. Diabetic retinopathy is one of the most common microvascular complications of T2DM and is closely associated with other complications such as cardiac autonomic neuropathy (CAN) and diabetic cardiomyopathy (DCM). A notable multicenter study conducted in Denmark (the DAN-Study) investigated the prevalence of CAN in DR patients across outpatient clinics and examined its relationship with T1DM and T2DM [136]. The findings revealed a significantly higher prevalence of CAN in T2DM patients with DR (35%). Furthermore, strong correlations were observed between CAN and critical risk markers of DR and DCM, including proliferative retinopathy, macroalbuminuria, and peripheral neuropathy. While this study provided valuable insights into the interconnected nature of DR and autonomic dysfunction, it did not investigate the direct effects of taVNS on DR or other microvascular outcomes. To date, no known studies have systematically evaluated the impact of taVNS on DR progression or its potential in mitigating microvascular complications associated with T2DM.

Renal Microvascular Disease and taVNS. Diabetic nephropathy is a significant microvascular complication of T2DM, characterized by progressive kidney damage that can lead to chronic kidney disease (CKD). Recent evidence highlights the potential of taVNS in ameliorating renal outcomes in T2DM. A meta-analysis revealed that taVNS treatment significantly slows the decline in estimated glomerular filtration rate (eGFR), reduces albuminuria progression, and improves overall renal outcomes in patients with T2DM [137]. As an emerging therapeutic modality, taVNS has shown promise when used as an adjunct therapy alongside sodium-glucose co-transporter-2 inhibitors (SGLT2i), a novel class of glucose-lowering agents. The combination of taVNS and SGLT2i has demonstrated synergistic benefits, including reductions in both cardiovascular and renal adverse outcomes among patients with T2DM, heart failure (HF), and nephropathy [138]. These findings underscore the potential of taVNS to modulate systemic and renal microvascular inflammation, contributing to better renal and cardiovascular health.

taVNS and Cardiac Fibrosis. The effects of taVNS on cardiac fibrosis have been investigated in preclinical studies, particularly in models of cardiotoxicity. In a study using a doxorubicin-induced cardiotoxicity model, tragus-based taVNS was shown to restore autonomic balance, improve cardiac function, and reduce interstitial and perivascular fibrosis. Additionally, taVNS attenuated myocyte apoptosis and ameliorated mitochondrial dysfunction compared to the untreated doxorubicin group [133]. These findings highlight the therapeutic potential of taVNS in addressing cardiac fibrosis, a hallmark of diabetic cardiomyopathy (DCM) and other cardiac complications of T2DM. However, further studies are needed to elucidate the relationship between microvascular dysfunction, fibrotic remodeling, and clinical outcomes after taVNS therapy in T2DM patients.

Data from Experimental Animal Models. Animal studies provide valuable mechanistic insights into the effects of taVNS on a microvascular and systemic level. A recent systematic review examined the impact of taVNS in preclinical models, analyzing data from eight studies conducted between 2015 and 2022 that included a total of 391 animal models [139]. The findings showed that taVNS significantly reduced neurological deficits and infarct size in stroke models. Additionally, taVNS was associated with enhanced markers of neuroplasticity, including increased microcapillary density, proliferation of CD31, and elevated brain-derived neurotrophic factor (BDNF) protein levels and RNA expression. Although this research focused on stroke models, the observed improvements in microvascular integrity and neuroplasticity suggest potential broader applications of taVNS in conditions such as T2DM, where microvascular dysfunction is a prominent feature. These preclinical results support the need for further research into the mechanisms and clinical utility of taVNS in treating T2DM-associated microvascular disease.

#### 3.3.2. Open Questions

##### Exploration of taVNS in Relation to T2DM-Associated HF—Is Clinical Translation Possible?

The progression of CVDs associated with T2DM, including atherosclerosis, hypertension, atrial fibrillation, and HF with preserved ejection fraction (HFpEF), is heavily influenced by autonomic dysfunction, such as heightened sympathetic nerve activity and chronic inflammation [140,141,142,143]. Over the past three decades, there has been a growing body of research investigating the efficacy of auricular vagus nerve stimulation (taVNS) as a therapeutic approach for various CVDs [140,144]. Studies have employed a range of taVNS methodologies, such as acupuncture, electrostimulation, and direct vagus nerve modulation [140,145,146]. Stimulation has primarily been applied to auricular regions such as the cymba concha and tragus [143,144]. Despite this body of work, definitive evidence regarding the protective effects of taVNS on T2DM-related HF remains inconclusive [147].

The mechanistic underpinnings of taVNS in the context of HF associated with T2DM are poorly understood, and scoping reviews on its potential benefits are sparse. Most current studies describe significant systemic effects observed in clinical settings but lack detailed mechanistic insights. This creates a disconnect between preclinical findings and clinical applications, making it difficult to ascertain the translational potential of taVNS for managing T2DM-related HF.

Preclinical research has demonstrated the cardioprotective effects of various neuromodulation techniques in HF models, with taVNS showing promise in animal studies [148]. However, clinical trials in patients with HF and reduced ejection fraction (HFrEF) have yielded inconsistent results [149]. For instance, the INOVATE-HF trial, a pivotal study assessing the impact of autonomic modulation devices on HF outcomes, found no evidence that vagus nerve stimulation (VNS) reduced mortality or cardiovascular events in patients with chronic HFrEF [150]. Conversely, alternative approaches, such as low-level tragus stimulation (LLTS), have shown potential in HFpEF, a condition often marked by low-grade systemic inflammation. LLTS was associated with reductions in inflammatory cytokines, macrophage infiltration, and myocardial fibrosis, as well as improvements in quality of life and life expectancy [148,151,152,153]. These mixed findings highlight the need for further research to determine the specific contexts in which taVNS may be effective for T2DM-associated HF.

##### Blood Pressure Regulation in T2DM and taVNS

Blood pressure dysregulation is a common complication in T2DM, contributing to increased cardiovascular risk. However, the effect of taVNS on blood pressure regulation in T2DM remains uncertain. A systematic review conducted according to PRISMA guidelines found no significant impact of taVNS on systolic blood pressure compared to control groups [142].

## 4. T2DM and the VN: Conclusions and Future Research Directions

T2DM, regardless of its subtype, is characterized by chronic hyperglycemia, which can lead to a wide array of complications if left inadequately managed. These include CVD, diabetic kidney disease (DKD), neuropathy, and other significant microvascular and macrovascular conditions. Persistent hyperglycemia damages blood vessels, significantly increasing the risk of heart attacks, strokes, and other cardiovascular events. Consequently, CVD remains a leading cause of morbidity and mortality among individuals with T2DM.

**Figure 6:**
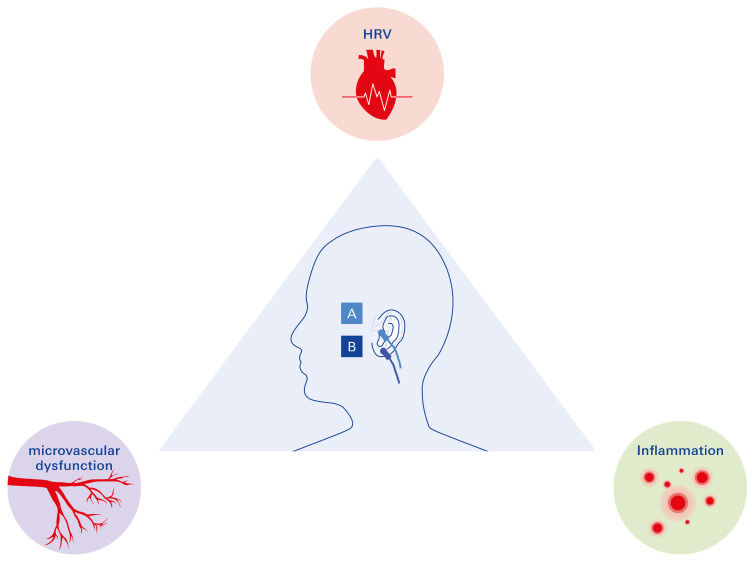
taVNS: a promising approach for managing diabetic complications. The vagus nerve (VN) plays a central regulatory role in critical factors associated with T2DM: (1) heart rate variability (HRV), (2) microvascular dysfunction, and (3) chronic inflammation. These factors can be positively modulated through vagal stimulation via transcutaneous auricular vagus nerve stimulation (taVNS). The blue clamps in the illustration denote anatomical sites of stimulation: A indicates the active stimulation site at the left tragus targeted by the taVNS device, while B represents the sham control stimulation site.

The vagus nerve (VN) has emerged as a key regulatory component in the management of diabetes, offering numerous avenues for exploration as a therapeutic target. The potential of VN modulation in treating T2DM is exemplified by interventions such as HRV-based monitoring, transcutaneous auricular vagus nerve stimulation (taVNS), and HRV biofeedback. These approaches show promise in leveraging the VN’s capacity to regulate autonomic balance and mitigate inflammation. As illustrated in Figure 6, VN modulation could lead to clinically safe changes in the LF/HF ratio of HRV, likely through increased vagal activity, which could positively impact the inflammatory and metabolic dysregulation associated with T2DM.

In the context of T2DM-related CVD, taVNS has shown some clinical evidence of benefit, particularly in the treatment of heart failure with preserved ejection fraction (HFpEF) [153]. Emerging data from studies on atrial fibrillation further support its potential efficacy. These benefits are primarily attributed to the modulation of systemic inflammation rather than direct effects on cardiac rhythm. This aligns with the growing body of evidence that systemic diseases with inflammatory pathogenesis, such as T2DM, could be ideal candidates for VN-targeted therapies [140,154]. However, while preclinical studies demonstrate encouraging results in reducing inflammation and restoring autonomic balance, the translation to consistent benefits in human trials has been limited [126,140]. Optimizing stimulation parameters, identifying effective stimulation sites, and stratifying patients into responders and non-responders may enhance the therapeutic impact of taVNS.

Despite its potential, taVNS has not yet been established as a standard treatment modality for T2DM. Preliminary findings are promising, but further research is essential to validate these outcomes, understand the mechanisms underlying its effects, and define the technology’s long-term safety and efficacy. Future studies should focus on refining delivery strategies, optimizing treatment protocols, and tailoring interventions to individual patient profiles. A critical objective for upcoming research is to elucidate the precise influence of VN modulation on the onset and progression of T2DM and its complications.

In conclusion, while taVNS and other VN-targeted approaches offer exciting possibilities, their full potential in T2DM management has yet to be realized. Bridging the gap between preclinical success and clinical efficacy remains a key challenge. However, with continued investigation, VN modulation could represent a transformative approach in the treatment of T2DM and its associated complications.

## Data Availability

Not applicable.

## Abbreviations

ABNV	auricular branch of the nervus vagus
AGE	advanced glycation end products
AF	atrial fibrillation
Ang-1	angiopoietin-1
BDNF	brain-derived neurotrophic factor
CAD	coronary artery disease
CAN	cardiac autonomic neuropathy
CD31	platelet endothelial cell adhesion molecule
CVD	cardiovascular disease
CCR2	CC motif chemokine receptor 2
DCM	diabetic cardiomyopathy
DR	diabetic retinopathy
DM	diabetes mellitus
ECG	electrocardiogram
GAN	great auricular nerve
HbA1c	hemoglobin A1c
HF	heart failure
HFrEF	heart failure reduced ejection fraction
HFpEF	heart failure preserved ejection fraction
HRV	heart rate variability
iBRB	inner blood retinal barrier
IL-1β	interleukin-1 beta
LLTS	low-level transcutaneous electrical stimulation of the tragus
MCP-1	monocyte chemoattractant protein-1
MMP	matrix metalloproteinase
oxLDL	oxidized low-density lipoprotein
PWA	P-wave alternans
NF-κB	nuclear factor ‘kappa-light-chain-enhancer’ of activated B-cells
RAAS	renin–angiotensin–aldosterone system
ROS	reactive oxygen species
SGLT2i	sodium-glucose transporter-2 inhibitor
taVNS	transcutaneous auricular vagus nerve stimulation
tVNS	transcutaneous vagus nerve stimulation
T1DM	type 1 diabetes mellitus
T2DM	type 2 diabetes mellitus
TNF-α	tumor necrosis factor α
VEGF	vascular endothelial growth factor
VNS	vagus nerve stimulation
